# Untangle the knot: Soybean MLH1 in meiotic recombination

**DOI:** 10.1093/plphys/kiae266

**Published:** 2024-05-08

**Authors:** Prateek Jain

**Affiliations:** Assistant Features Editor, Plant Physiology, American Society of Plant Biologists; Department of Biology, The University of North Carolina at Chapel Hill, NC 27599-3280, USA

Climate change and global food demand require sustainable efforts to improve crop productivity and yields. The classical plant breeding approach harnesses the advantages of meiotic recombination or crossing over (CO) for crop development and trait improvement. During meiotic recombination, homologous chromosomes exchange genetic segments through pairing, synapsis, and recombination to generate genetic diversity. Several CO protein complexes and families like ZMM complex (Zipper 1-4, Mut S homolog 4-5, and meiotic recombination 3), MutLγ protein family (MLH1 and MLH3), and others ensure the error-free meiosis and uniform chromosome distribution in daughter cells without genetic anomalies (Wang and Copenhaver 2018) (Fig. A).

**Figure:**
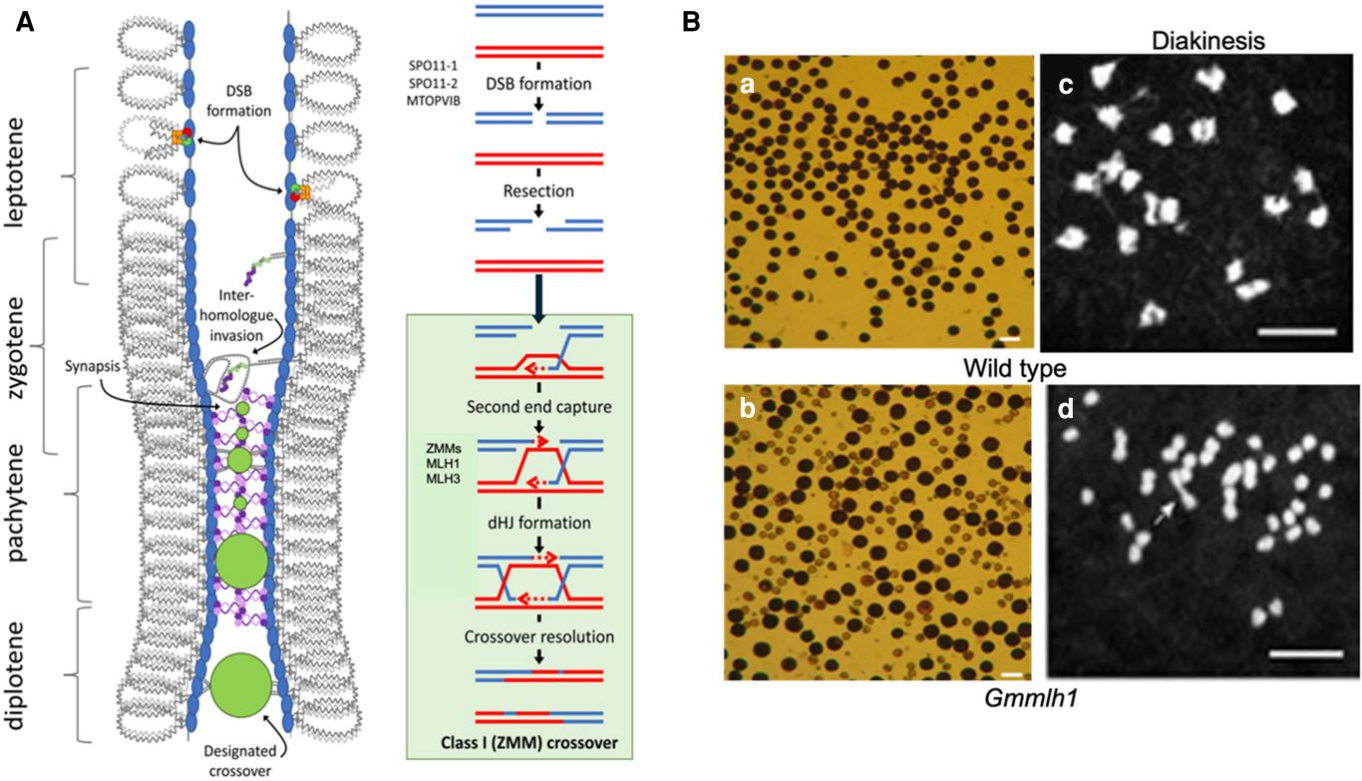
**A)** Schematic representation of DNA repair pathway/meiotic recombination in plants (image reprinted and modified from Lloyd A. 2023) (Lloyd 2023). **B)** Pollen grains and chromosomes in pollen mother cells of wild type and *Gmmlh1* mutant. a and c, Wild-type pollen grains and chromosome bivalents. b and d, *GmmIh1* aborted pollen grains and univalent (mark by arrow) chromosomes. Scale bar = 10 *μ*m.

Genetic COs are tightly regulated by 2 pathways. The first is class I interference sensitive, which is controlled by the ZMM and MutLγ protein family and regulates 85% to 90% of COs (Jackson et al. 2006). The remaining COs are governed by a class II interference-insensitive pathway, which is directed by MUS81 and FANCD2 (Kurzbauer et al. 2018). Because genetic recombination is key to diversity in nature, how proteins regulate COs is an active question in yeast, humans, and plants, and understanding it can be particularly beneficial for enhancing breeding of crop plants (Keeney et al. 1997; Wang and Copenhaver 2018).

In this issue of *Plant Physiology*, Wu et al. (2024) targeted soybean, an economically valuable crop that is highly desirable for oil and protein content, and investigated the role of GmMLH1, a homologue of MutLy- protein family, during meiotic recombination (Wu et al. 2024). Expanding on their previous work on ethyl methanesulfonate mutagenized soybean cultivar Williams 82, the authors identified a mutant line with fertility defects in both male and female gametes, which they named *Gmfms1* (*Glycine max female and male sterility 1*) (Feng et al. 2019).

With experimental validation that *Gmfms1* has abnormal gamete phenotypes, the authors sought out the responsible causative gene. They crossed the *Gmfms1* mutant with an elite Chinese soybean cultivar, Hedou 12, and observed a 3:1 segregation ratio in the F_2_ generation. The finding confirmed that a single recessive gene is responsible for the mutant phenotype in *Gmfms1*. To identify the responsible gene, Wu et al. (2024) selected the F_2_ plants with lesser than 2 seeds per plant and performed bulked segregant analysis sequencing. The sequenced mutants were mapped and compared against the wild type, which helped to identify a single nucleotide mutation in Glyma.04G254900. Moreover, Glyma.04G25490 was found to be similar to Arabidopsis *MLH1*(*AtMLH1*) and hence was labeled as *GmMLH1* in the present study. To determine whether GmMLH1 has a universal role across soybean cultivars, the authors knocked out the *GmMLH1* by CRISPR/Cas9 in soybean cultivar Dongnong 50. The CRISPR-edited mutants plants exhibit reduced fertility with deformed pollen grains (Fig. B) that confirms the direct role of GmMLH1 in sexual reproduction (Wu et al. 2024) and that the *GmMLH1* gene is responsible for the phenotype of *Gmfms1*, which is renamed as the *Gmmlh1* mutant.

Having confirmed that GmMLh1 plays a role similar to AtMLH1, which forms heterotypic interaction with AtMLH3 to resolve double Holliday junctions into COs during meiosis (Wang and Copenhaver 2018), Wu et al. (2024) investigated meiosis I and specifically prophase I to understand the chromosome architecture and distribution in the nucleus. The chromosomes in wild type are linked and present as bivalent, whereas in *Gmmlh1*, chromosomes are separated from one other as univalent and randomly distributed throughout the nucleus, which confirms abnormal class I pathway (interference-sensitive crossover pathway) (Fig. B) (Cannavo et al. 2020; Wu et al. 2024). Fewer chromosome bivalents indicate potential COs loss and accounts for the fertility defects in the *Gmmlh1* mutant. Furthermore, the dimeric interaction between GmMLH1 and GmMLH3 was validated by split-luciferase complementation, bimolecular fluorescence complementation, and in-vitro pull-down assays.

In addition to meiosis, the authors were also interested to understand the role of GmMLH1 in somatic DNA damage repair, as MLH1 was previously reported to be involved in both meiotic and mitotic recombination (Dai et al. 2021). The authors treated *Gmmlh1* seedlings with Mitomycin C (MMC), a chemical that forms interstrand cross-link adducts and produces DNA strand break (Wu et al. 2024). MMC-treated *Gmmlh1* seedlings were found to have a higher frequency of distorted and abnormal mitotic cells compared with wild type, confirming its role in both mitotic and meiotic recombination.

Overall, the research work of Wu et al. (2024) establishes the role of GmMLH1 in DNA break repair pathway and its involvement in both mitosis and meiosis in soybean (Wu et al. 2024). Gmmlh1 mutant was found to have abnormal pollen and aborted embryo sac phenotypes, signifying that it is interfering with meiotic recombination and restricting the gamete fertility. This study will help to understand the complex molecular pathways like recombination and employ synthetic biology tools to develop crops with better traits and higher yields.
